# Transcriptional Upregulation of NLRC5 by Radiation Drives STING- and Interferon-Independent MHC-I Expression on Cancer Cells and T Cell Cytotoxicity

**DOI:** 10.1038/s41598-020-64408-3

**Published:** 2020-04-30

**Authors:** Lauren K. Zebertavage, Alejandro Alice, Marka R. Crittenden, Michael J. Gough

**Affiliations:** 1 Earle A. Chiles Research Institute, Providence Portland Medical Center, Portland, OR 97213 USA; 2 Molecular Microbiology and Immunology, Oregon Health and Science University, Portland, OR 97239 USA; 3 The Oregon Clinic, Portland, OR 97213 USA

**Keywords:** Cancer, Tumour immunology, Adaptive immunity, Immunotherapy, T cells, CD8-positive T cells

## Abstract

Radiation therapy has been shown to enhance the efficacy of various T cell-targeted immunotherapies that improve antigen-specific T cell expansion, T regulatory cell depletion, or effector T cell function. Additionally, radiation therapy has been proposed as a means to recruit T cells to the treatment site and modulate cancer cells as effector T cell targets. The significance of these features remains unclear. We set out to determine, in checkpoint inhibitor resistant models, which components of radiation are primarily responsible for overcoming this resistance. In order to model the vaccination effect of radiation, we used a *Listeria monocytogenes* based vaccine to generate a large population of tumor antigen specific T cells but found that the presence of cells with cytotoxic capacity was unable to replicate the efficacy of radiation with combination checkpoint blockade. Instead, we demonstrated that a major role of radiation was to increase the susceptibility of surviving cancer cells to CD8+ T cell-mediated control through enhanced MHC-I expression. We observed a novel mechanism of genetic induction of MHC-I in cancer cells through upregulation of the MHC-I transactivator NLRC5. These data support the critical role of local modulation of tumors by radiation to improve tumor control with combination immunotherapy.

## Introduction

It is clear that radiation therapy (radiotherapy, RT) has significant immune modulatory effects and is capable of unleashing potent anti-tumor CD8+ T cell responses^1–6^. The ability of radiotherapy to enhance adaptive immune responses has further been highlighted by the ability of radiation to enhance the therapeutic efficacy of PD-1/PD-L1 checkpoint blockade in preclinical models^6–10^. While interest in abscopal effects, or those observed outside of the field of irradiation, have increased in part due to the observation that radiation can act as an *in situ* vaccine^10,11^, recent studies by our group and others have determined that combination radiation and checkpoint blockade therapy requires pre-existing T cell responses to control tumors^1,12^. Given the strong interest in using existing therapies such as radiation to enhance αPD-1/αPD-L1 responses in human cancers, it is critical to understand the mechanisms by which RT is improving outcomes to better inform treatment of patients^13,14^.

In this study, we aimed to determine the mechanisms by which radiation overcomes αPD-L1 therapeutic resistance using murine models of pancreatic cancer expressing model antigens. Here, we were able to dissect out the role of vaccination effects, T cell trafficking, and cancer cell phenotype modifications and we found that while radiation was able to boost tumor-specific CD8+ T cell responses, vaccine effects were not sufficient to recapitulate the efficacy of radiotherapy with checkpoint blockade. We found that in our model radiation did not improve trafficking or retention of tumor-reactive CD8+ T cells to tumors. However, experiments *in vitro* and *in vivo* indicated that alterations in cancer cell phenotypes, particularly by upregulation of MHC-I surface expression, are sufficient to enhance control of tumors by antigen-specific CD8+ T cells. Finally, we observed a novel mechanism of transcriptional regulation of MHC-I expression on tumor cells by expression of the MHC-I transactivator NLRC5 (NOD-like receptor C5) and found that expression of NLRC5 by cancer cells enhanced cytotoxic cytokine production by CD8+ T cells.

## Results

**Figure 1:**
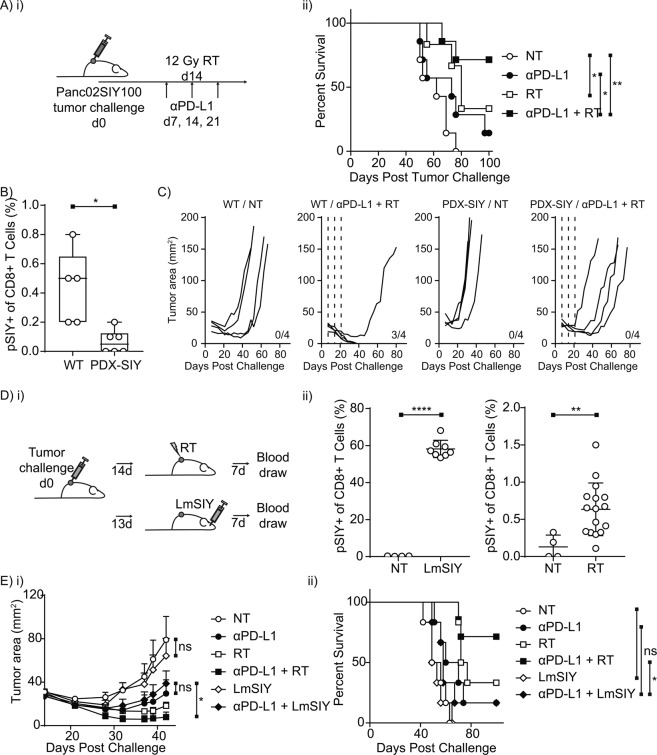
Model antigen-reactive CD8+ T cells are necessary but not sufficient to induce regression of Panc02SIY100 tumors. (**A**) (i) treatment schema, (ii) overall survival of treatment groups: no treatment (NT), αPD-L1 alone (αPD-L1), radiation alone (RT) and combination αPD-L1 and radiation (αPD-L1 + RT). (**B**) SIYRYYGL-pentamer (pSIY)- binding CD8+ T cells quantified from blood at day 7 following Panc02SIY tumor implantation in wild-type (WT) or SIYRYYGL-tolerant (PDX-SIY) mice. (**c**) tumor growth from mice implanted with Panc02SIY100 tumors and treated as in (**A**). (**D**) (i) treatment schema, (ii) pSIY-binding CD8+ T cells quantified from blood a day 7 following *LmSIY* vaccination or tumor irradiation. (**E**) (i) average tumor growth from mice implanted with Panc02SIY100 tumors and treated with αPD-L1 and RT as described in A or LmSIY at day 14 (ii) overall survival of treatment groups. Key: *p < 0.05; **p < 0.01; ****p < 0.0001; ns = not significant.

In order to determine if the generation of tumor-specific CD8+ T cells by tumor irradiation was sufficient to induce overcome αPD-L1 checkpoint blockade therapeutic resistance, we used the murine Panc02 model of pancreatic adenocarcinoma^15^ expressing a fusion of eGFP and the model antigen SIYRYYGL (SIY) and purified for high expression of antigen (Panc02SIY100)^16^. Subcutaneous Panc02SIY100 tumors in C57BL/6 mice are resistant to αPD-L1 checkpoint blockade (median survival NT 62d *vs*. anti-PD-L1 73d p = 0.1771), have a low cure rate with a single dose of RT (12 Gy) (median survival NT*vs*.RT 80d p = 0.0108), and a cure rate that is markedly enhanced by the combination of RT and αPD-L1 checkpoint blockade (Fig. 1A). In this model there is not a therapeutic synergy since while the combination of RT and anti-PD-L1 increase cure rates they are not significantly different by Logrank test from RT alone (median survival RT + anti-PD-L1 *undefined vs*. anti-PD-L1 p = 0.0306; *vs*. RT p = 0.2460). However, it is important that combination is not effective in mice tolerant to the model antigen SIY (PDX-SIY) (Fig. 1A–C), indicating that effective tumor cure induced by RT + anti-PD-L1 to Panc02SIY100 is entirely dependent on CD8+ T cells responsive to the model antigen.

To test whether a large number of model antigen-reactive CD8+ T cells was sufficient to replicate the efficacy of RT in Panc02SIY100, we used a live-attenuated *Listeria monocytogenes* vaccine expressing SIY (*LmSIY*) to generate large numbers of tumor-reactive CD8+ T cells. While radiation therapy was able to significantly increase the number of SIY-specific T cells in circulation (p < 0.01), *LmSIY* vaccination was more than an order of magnitude more effective at generating SIY-specific T cells (Fig. 1D). Despite these increases in SIY-reactive CD8+ T cells compared to radiation alone, vaccination did not significantly improve survival or slow tumor growth either alone or in combination with anti-PD-L1 (Fig. 1D,E). Similarly, in an immune competent animal, adding vaccination with *LmSIY* to RT did not improve tumor control in the absence of anti-PD-L1 (Supplemental Fig. 1). These results indicate that potent anti-cancer vaccination strategies cannot replicate the efficacy of RT combined with αPD-L1, suggesting additional radiation-induced changes other than the induction of tumor-specific CD8+ T cells are required to control tumors.

**Figure 2:**
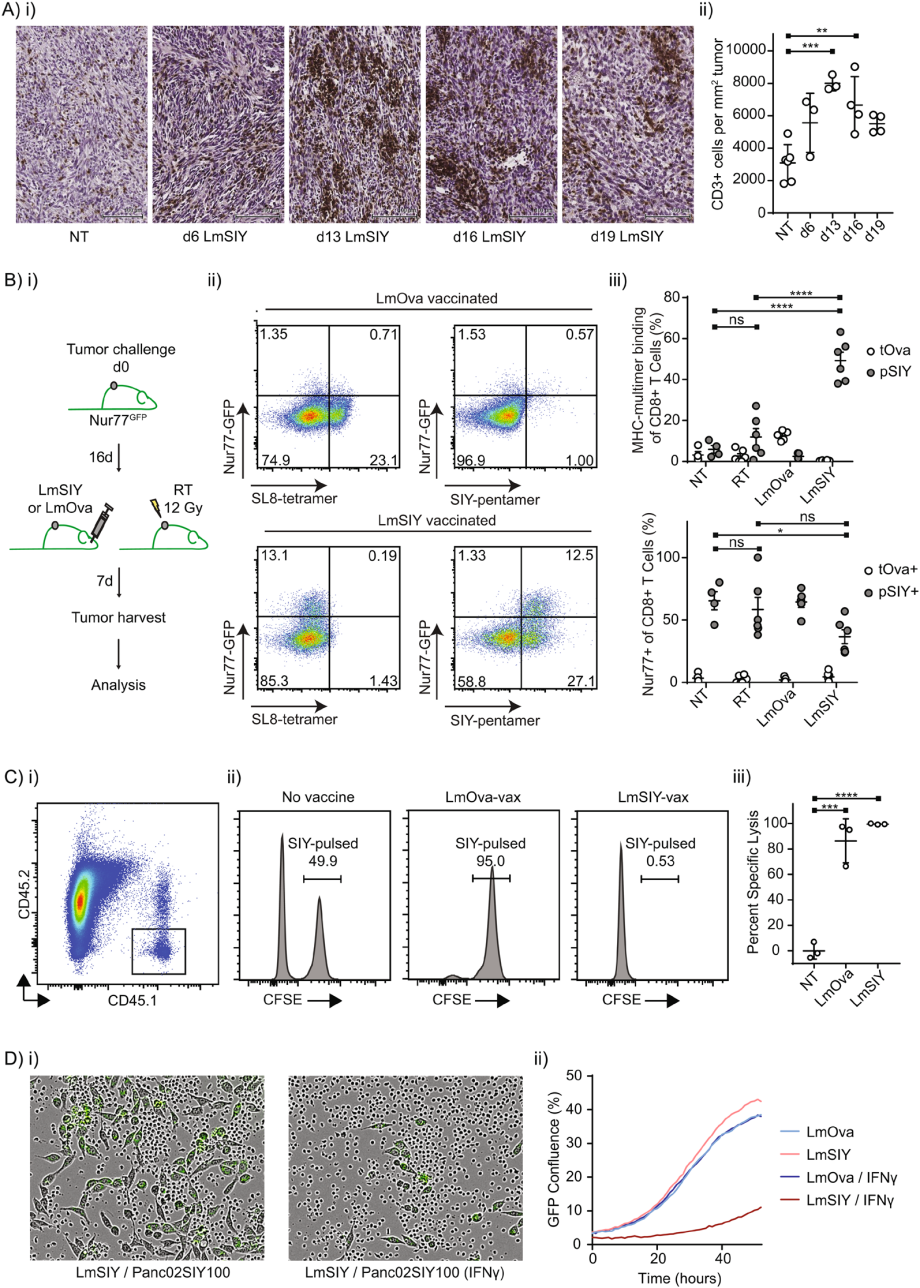
*Listeria*-induced CD8+ T cells are cytotoxic *in vivo* but are poorly effective at controlling tumor growth in the absence of interferon pretreatment. (**A**) (i) immunohistochemistry of CD3 infiltration from tumor sections from mice not treated (NT) or treated with *LmSIY* at indicated times following tumor challenge (d6, d13, d16 and d19), (ii) quantification of CD3+ infiltration by immunohistochemistry. (**B**) (i) representative dot plot of splenocytes, (ii) representative histograms of CFSE-labeled CD45.1^+^ cells from unvaccinated, *LmOva* vaccinated or *LmSIY* vaccinated mice, (iii) quantification of percent specific lysis where NT and *LmSIY* reflects lysis of SIYRYYGL-pulsed cells and *LmOva* reflects lysis of SIINFEKL-pulsed cells. (**C**) (i) treatment schema, (ii) representative dot plots of tumor-infiltrating CD8+ T cells, (iii) quantification of MHC-multimer binding of tumor-infiltrating CD8+ T cells where tOva represents SIINFEKL tetramer binding cells and pSIY represents SIYRYYGL pentamer binding cells, (iv) quantification of Nur77-GFP^+^ cells of tumor-infiltrating CD8+ T cells where tOva represents SIINFEKL tetramer binding cells and pSIY represents SIYRYYGL pentamer binding cells. (**D**) (i) representative Incucyte images of Panc02SIY100 cells untreated or pretreated for 24 hours with IFNγ and cocultured with splenocytes from *LmSIY*-vaccinated mice, (ii) growth chart of Panc02SIY100 cancer cells following coculture with splenocytes from *LmOva-* or *LmSIY*- vaccinated animals. Key: *p < 0.05; **p < 0.01; ***p < 0.001; ****p < 0.0001; ns = not significant.

We designed a series of experiments to test the trafficking and functionality of *Listeria*-induced CD8+ T cells to determine whether this could account for the inferior outcomes we observed with *LmSIY* and αPD-L1. To determine whether the failure of *Listeria*-induced CD8+ T cells to cure was due to failure to traffic to tumors, we examined tumors post-RT by immunohistochemistry (IHC). T cells were found in greater numbers following vaccination when compared to untreated controls and formed distinct clusters at peak infiltration, suggestive of *in situ* activation (Fig. 2A). Notably, T cell infiltration following tumor irradiation detected by IHC was comparable to numbers following vaccination with *LmSIY* (data not shown). In order to test the *in vivo* functionality of *Listeria*-induced CD8+ T cells, we performed an *in vivo* CTL assay, where target or irrelevant peptide-pulsed congenic splenocytes were co-transferred into vaccinated animals. As expected, *LmSIY* vaccination selectively depleted SIY-pulsed cells, and the control *LmOva* vaccination selectively depleted SIINFEKL-pulsed cells (Fig. 2B), indicating that *Listeria* vaccines formed functional antigen-specific CD8+ T cell cytotoxic immunity for both *LmSIY* and control *LmOva*. To test whether vaccine-generated T cells recognized antigen within tumors, transgenic Nur77^GFP^ reporter mice^17^ were implanted with SIY-expressing tumors and either vaccinated or treated with radiation therapy (Fig. 2Ci). T cells from Nur77^GFP^ mice become fluorescent following ligation of the T cell receptor with its cognate antigen and lose signal over a narrow window of time (data not shown). Tumors harvested from Nur77^GFP^ mice following *LmSIY* or *LmOva* vaccination resulted in the accumulation of SIY-specific or SIINFEKL-specific T cells in the tumor, respectively (Fig. 2Cii). As expected, SIY-specific T cells actively recognized antigen in the tumor as determined by expression of Nur77-GFP, while SIINFEKL-specific T cells did not (Fig. 2Ciii). Importantly, Nur77-GFP expression in SIY-specific T cells was not significantly different across treatments (Fig. 2Civ), indicating that T cells generated by vaccination recognize tumor antigen at similar rates to radiation-generated and existing tumor-infiltrating SIY-specific T cell populations. To test whether *Listeria*-induced CD8+ T cells were capable of controlling antigen-matched cancer cells, CD8+ T cells were harvested from non-tumor-bearing animals vaccinated with *LmSIY* or *LmOva* and cocultured with Panc02SIY100. Cancer cell growth was monitored microscopically *in vitro* over time with green fluorescence as a proxy for cancer cell confluence (Fig. 2Di). Significantly, *LmSIY*-induced splenocytes were not capable of suppressing Panc02SIY100 growth *in vitro* unless cancer cells were pretreated with interferon gamma (IFNγ) prior to coculture (Fig. 2Dii). Taken together, these results indicate that *LmSIY*-induced CD8+ T cells are cytotoxic, effectively traffic to SIY-expressing tumors and recognize antigen in the tumor; however, Panc02SIY100 cells are intrinsically resistant to killing by antigen-specific CD8+ T cells in the absence of phenotypic modifications induced here by IFNγ.

**Figure 3:**
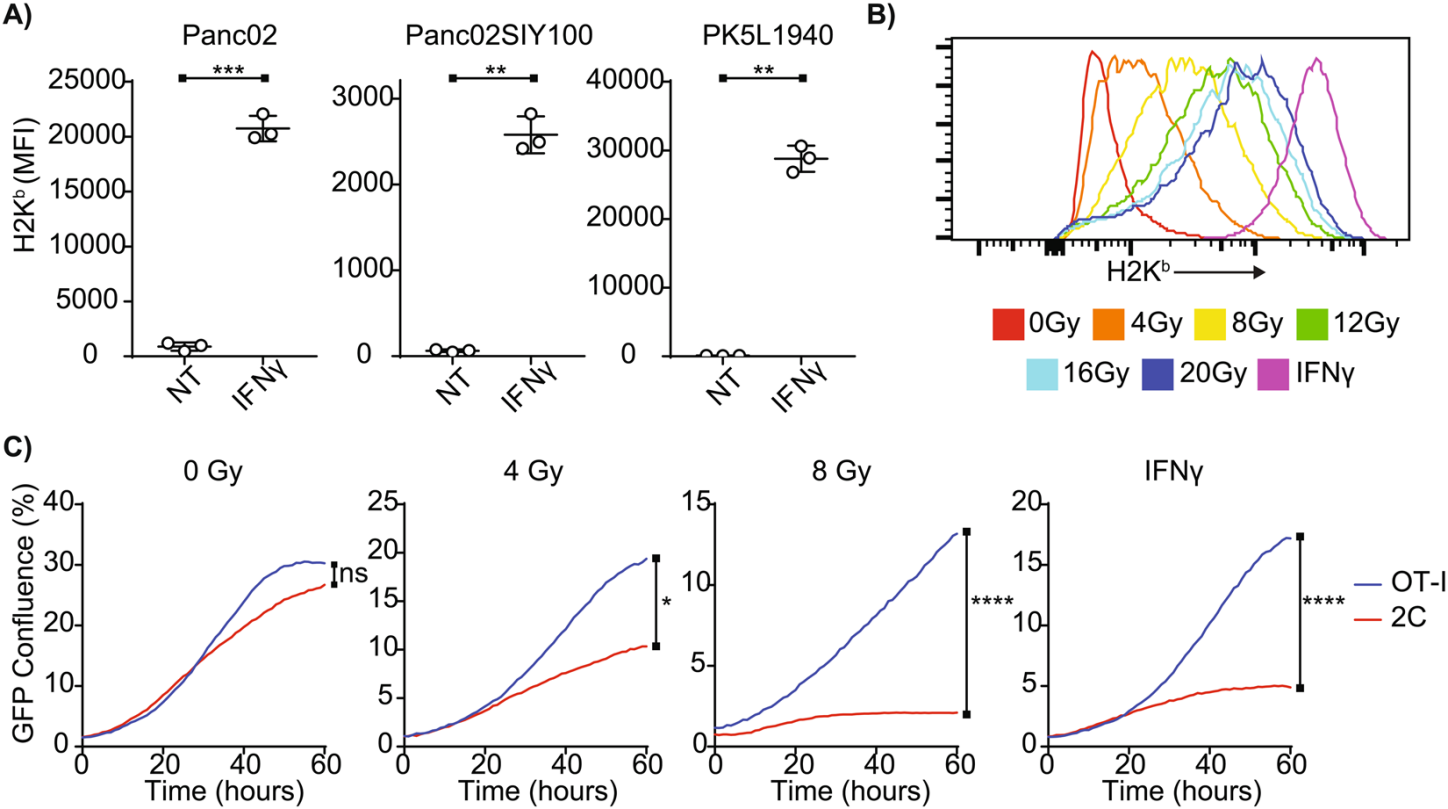
Radiation increases MHC-I expression and improves susceptibility of cancer cells to control by antigen-specific CD8+ T cells. (**A**) Quantification of MHC-I expression by flow cytometry of pancreatic cancer cell lines with and without 72 hours of IFNγ stimulation. (**B**) Representative histogram of MHC-I expression of Panc02SIY100 cancer cells 72 hours following irradiation at indicated doses. (**C**) growth charts of Panc02SIY100 cancer cells following pretreatment with *in vitro* irradiation or IFNγ and during coculture with *in vitro* activated OT-I or 2 C T cells. Key: *p < 0.05; **p < 0.01; ****p < 0.0001; ns = not significant.

These experiments led us to consider phenotypic modifications induced by IFNγ stimulation that are shared by irradiation, among which increased cell surface presentation of MHC-I (Fig. 3A) is of particular interest due to the essential role of antigen presentation in CD8+ T cell function. The pancreatic adenocarcinoma cell lines have very low basal MHC-I expression, but are responsive to IFNγ stimulation (Supplementary Fig. 2). RT is also a known modulator of MHC-I expression and similarly, we observed dose-dependent increases in MHC-I on tumor cell surfaces by RT (Fig. 3B). Similar to IFNγ pretreatment, we observed that irradiating tumor cells was sufficient to induce modifications within the cancer cells to allow control *in vitro* by tumor antigen-specific CD8+ T cells (Fig. 3C). IFNγ was more effective than radiation in inducing MHCI on the cancer cells (Fig. 3B), and we were not able to further increase the dose of radiation to attempt to amplify MHCI induction due to its cytotoxic effect on the cancer cells. Moreover, IFNγ was able to maximally induce MHCI on cancer cells and did not permit an additive effect from combining RT and IFNγ treatment *in vitro*. Based on these data, we hypothesized that irradiation improves cancer cell susceptibility to CD8+ T cell mediated killing by augmenting MHC-I expression.

**Figure 4:**
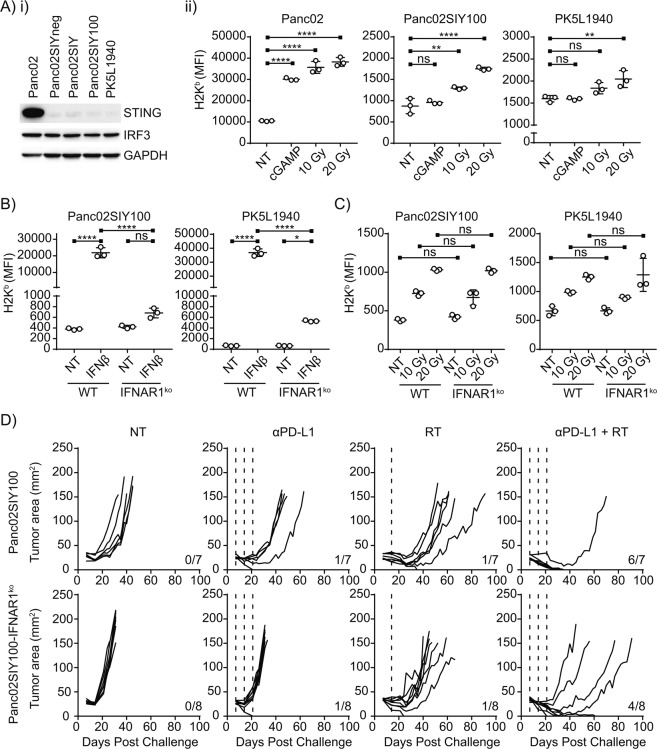
Type I interferon (IFN-I) responsiveness does not dictate MHC-I expression or clearance of Panc02SIY100 tumors. (**A**) Western blot of STING and IRF3 expression in pancreatic cell lines with GAPDH loading control. (**B**) flow cytometric MFI of MHC-I expression of pancreatic cell lines 72 hours after stimulation with 2′3′-cGAMP or irradiation *in vitro*. (**C**) quantification of MHC-I expression of pancreatic cell lines following 72 hours IFNβ stimulation. (**D**) quantification of MHC-I expression of pancreatic cell lines 72 hours following irradiation at indicated doses. (**E**) tumor growth of mice implanted with Panc02SIY100 or Panc02SIY100-IFNAR1^ko^ tumors and treated as in 1A. Key: *p < 0.05; **p < 0.01; ***p < 0.001; ****p < 0.0001; ns = not significant.

The mechanisms by which radiation induces MHC-I expression in cancer cells is not completely understood. Cancer cells are not thought to be sources of IFNγ, but MHC-I expression can be directly regulated by type I interferon (IFN-I) signaling and studies have indicated that host responses to IFN-I through the receptor IFNAR are essential to the anti-cancer CD8+ T cell response driven by radiotherapy^18^. Recently, a series of publications indicated that IFN-I secretion following RT might be initiated by cancer cells themselves responding to cytosolic DNA fragments activating cGAS/STING, a process negatively regulated by activation of the DNA exonuclease Trex1^19–21^. To test the role of cancer cell intrinsic STING signaling in MHC-I induction in cancer cells, cancer cell lysate was immunoblotted and we observed that, contrary to the parental cancer cell line Panc02, Panc02SIY100 expresses very low levels of STING protein (Fig. 4A). To determine whether this was unique to Panc02SIY100, we also tested pancreatic cancer cells derived from Pdx-Cre^+/−^Kras^(G12D)+/−^Trp53^(R172H)+/−^R26^LSL-LSIY/+^ mice, PK5L1940^1^ and found similarly low STING expression. We further observed by treating cancer cells lines *in vitro* with RT or the endogenous STING ligand 2′,3′-cGAMP that cell lines with low expression of STING protein were still able to upregulate MHC class I (MHC-I) in response to irradiation but not to STING ligand. In contrast, Panc02 was able to upregulate MHC-I in response to both treatment with 2′,3′-cGAMP and radiation (Fig. 4B). These results indicate that STING signaling in cancer cells is not required for RT-induced MHC-I modulation.

Previous work has indicated that cancer cell-derived IFN-I secreted following irradiation induces MHC-I upregulation on cancer cells^22^. To test whether alternate inflammatory sensors drive MHC-I upregulation via endogenous IFN-I production, we knocked out the IFN-I receptor (IFNAR) in cancer cell lines. IFNAR is a heterodimeric receptor consisting of two subunits, IFNAR1 and one of several distinct isoforms of IFNAR2^23^. Mutation of the IFNAR1 gene by CRISPR/Cas9 editing removed detectable IFNAR1 expression on the cell surface (Supplementary Fig. 3) and rendered cancer cells insensitive to exogenous IFNβ stimulation (Fig. 4B). The pancreatic adenocarcinoma cell lines exhibited poor basal expression of STAT1, though they upregulated STAT1 following IFNβ treatment in wild type, but not IFNAR1^ko^ cell lines (Supplementary Fig. 4). The addition of RT to IFNβ did not alter the response to IFNβ, and RT was not able to upregulate STAT1 without IFNβ, consistent with a lack of STING pathway activation (Supplementary Fig. 4). As expected, the response to IFNγ was unchanged in IFNAR1^ko^ cell lines. Significantly, IFNAR1^ko^ cell lines that are unable to upregulate MHC-I in response to exogenous IFNβ stimulation retain their ability to upregulate MHC-I in response to RT (Fig. 4C). To determine whether loss of cancer cell IFN-I signaling affected RT efficacy, we treated Panc02SIY100-IFNAR1^ko^ tumors with therapeutic doses of RT and αPD-L1 *in vivo* and found similar combined efficacy (Fig. 4D). Notably, overall survival in the combination therapy group was higher in mice bearing parental tumors than Panc02SIY100-IFNAR1^ko^, likely due to the more aggressive growth rate of cancer cells unable to respond to type I IFN. Tumors formed from cancer cells lacking IFNAR were analyzed for infiltrating immune cells by flow cytometry, and although these tumors were generally larger, there was not significant difference in the baseline number of the major immune cell populations, or SIY-specific CD8 T cells (Supplementary Fig. 5). Radiation therapy also resulted in similar effects on these immune cells following treatment of wild-type or IFNAR1^ko^ tumors (Supplementary Fig. 5), suggesting that the responsiveness of cancer cells to type I IFN does not greatly impact the tumor immune environment. Together, these data demonstrate that the endogenous cancer cell response to IFN-I is not required for upregulation of MHC-I by radiation or tumor control by radiation and αPD-L1 blockade.

**Figure 5:**
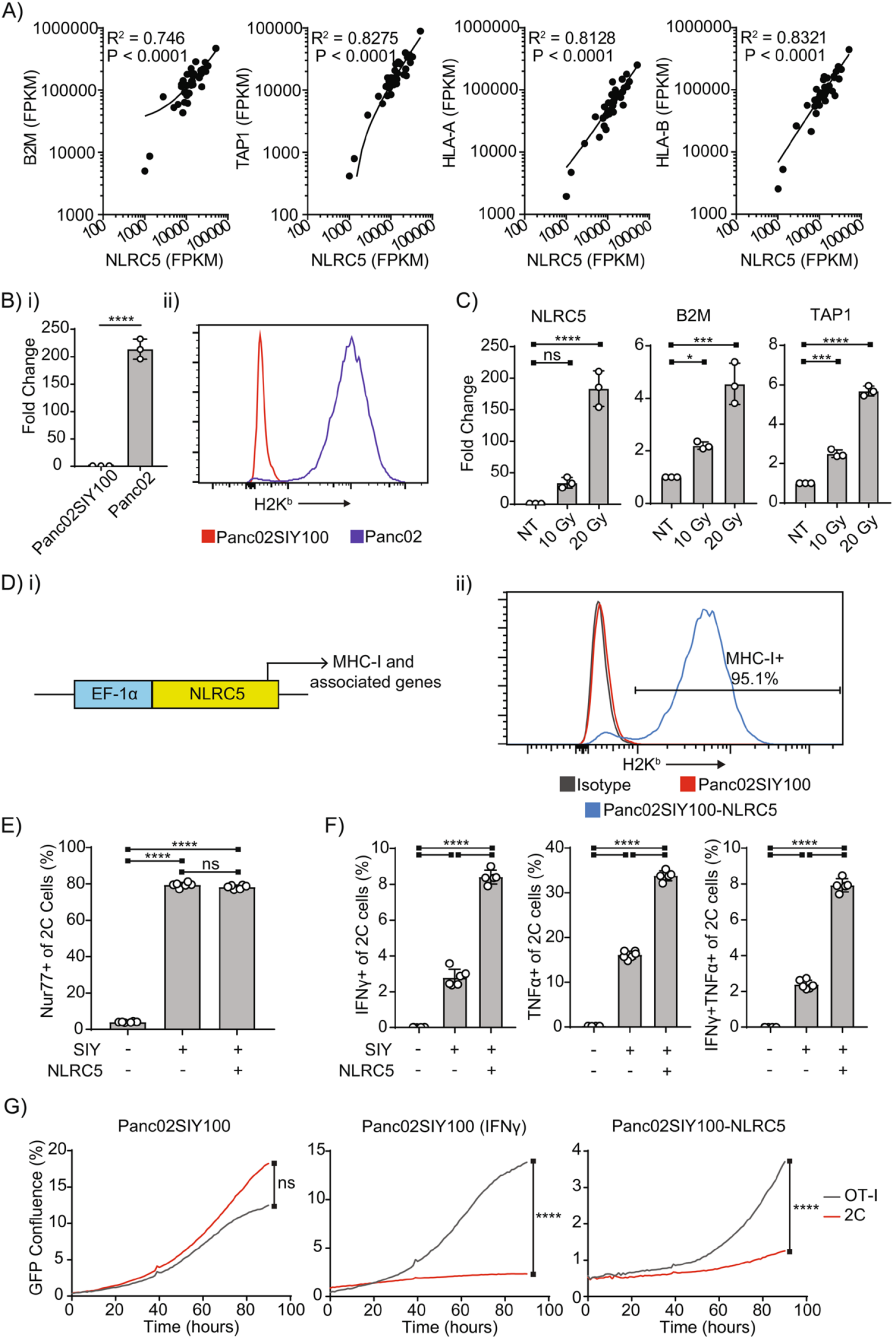
NLRC5 expression by tumors increases expression of MHC-I and improves tumor control by antigen-specific CD8+ T cells. (**A**) RNAseq data from pancreatic adenocarcinoma patient samples correlating NLRC5 expression with genes involved with antigen processing and presentation. (**B**) (i) qRT-PCR data comparing NLRC5 expression of untreated pancreatic cancer cell lines normalized to GAPDH expression, (ii) representative histogram comparing surface MHC-I expression of untreated pancreatic tumor cell lines. (**C**) qRT-PCR data comparing gene expression in Panc02SIY100 cells 72 hours following irradiation at indicated doses. (**D**) (**i**) NLRC5 gene construct, (**ii**) representative histogram comparing MHC-I expression in untreated Panc02SIY100 with and without induced NLRC5 expression. (**E**) quantification of Nur77-GFP expression of 2C^Nur77-GFP^ CD8+ T cells 24 hours after coculture with pancreatic cancer cell lines (Panc02SIYneg, Panc02SIY100, Panc02SIY100-NLRC5). (**F**) quantification of cytotoxic cytokine production by 2C cells 72 hours after coculture with pancreatic cancer cell lines (Panc02SIYneg, Panc02SIY100, Panc02SIY100-NLRC5). (**G**) growth charts of Panc02SIY100 with or without IFNγ pretreatment for 24 hours or Panc02SIY100-NLRC5 during coculture with *in vitro* activated OT-I or 2C T cells. Key: *p < 0.05; ***p < 0.001; ****p < 0.0001; ns = not significant.

A key genetic driver of MHC-I expression is the MHC Class I Transactivator (CITA, or NLRC5)^24–26^, a reported target of immune evasion in human cancers^27^. To determine whether this was true for human pancreatic cancers, we examined RNAseq gene expression data from 35 human patients with resectable pancreatic adenocarcinoma (Table 1) and found that NLRC5 expression tightly correlated with expression of MHC-I heavy chain HLA molecules, light chain β2 microglobulin (B2M) and TAP1 expression (Fig. 5A). This was also true when examining mRNA expression in patients analyzed as part of the TCGA Pancreatic Adenocarcinoma PanCancer Atlas^28^ as a validation cohort using cBioportal^29,30^ (Supplementary Table 1). In these patients, NLRC5 was highly correlated with HLA-B expression along with other key components of the antigen processing and presentation pathway. In our murine pancreatic cancer cell lines, we found that the parental cell line Panc02 had much higher expression of NLRC5 than the Panc02SIY100 subclone, correlating with its much higher basal expression of MHC-I (Fig. 5B). Baseline expression of NLRC5 was very low, close to the limit of detection (Supplementary Fig. 6), but we found that irradiation of Panc02SIY100 significantly increased transcription of NLRC5 and related genes B2M and TAP1 in a dose-dependent manner (Fig. 5C). NLRC5 is also upregulated following radiation therapy in cells lacking IFNAR1 (Supplementary Fig. 7), indicating that this mechanism is independent of IFN signaling in irradiated cells. Therefore, we believe that upregulation of NLRC5 represents a potentially novel mechanism of MHC-I regulation induced by radiation.

**Table 1:** Pancreatic adenocarcinoma RNASeq patient characteristics.

Category	Criteria	Number of patients
Sex	Male	19
	Female	16
T stage	ND	1
	1	1
	2	3
	3	28
	4	2
N stage	0	9
	1	26
M stage	0	35
	1	0
Age	Mean – 68 StDev - 10	Total 35

To determine whether constitutive NLRC5 expression was sufficient to restore MHC-I in Panc02SIY100, cells were transfected with a plasmid harboring the sequence for NLRC5 under the constitutive promoter eukaryotic translation elongation factor 1 alpha (EF-1α) and transfected cells were found to have very high basal levels of MHC-I expression (Fig. 5D). To determine the functional consequences of MHC-I induction by NLRC5 expression in cancer cells, Panc02SIY100-NLRC5 was cocultured with 2 C T cells from Nur77^GFP^ mice. Equivalent proportions of 2 C T cells recognized tumor antigen when cocultured with MHC-I^hi^ Panc02SIY100-NLRC5 compared to MHC-I^lo^ Panc02SIY100 based on GFP expression (Fig. 5E). Importantly, however, expression of NLRC5 by Panc02SIY100 significantly enhanced cytotoxic cytokine secretion by 2 C cells (Fig. 5F), indicating that while low MHC-I expression is not a barrier to recognition of tumors by cytotoxic CD8+ T cells, upregulation of MHC-I on target cells by NLRC5 expression significantly improves the ability of CD8+ T cells to functionally respond to the antigenic stimulation. In order to test whether cancer cell upregulation of MHC-I by NLRC5 is sufficient to explain the increased ability of CD8+ T cells to drive tumor clearance, we generated cancer cell lines with constitutively high levels of MHC-I. In *in vitro* T cell coculture experiments, expression of NLRC5 by Panc02SIY100 is sufficient to enhance the ability of activated 2C cells to control cancer cell growth (Fig. 5G). Taken together, these experiments demonstrate tight correlation between both endogenous and induced expression of NLRC5 and MHC-I expression in murine pancreatic cancer cell lines and in human patients and indicate that increased expression of NLRC5, as induced by irradiation, is sufficient to enhance cytotoxic activity by antigen-specific CD8+ T cells in response to antigenic stimulation by cancer cells.

## Discussion

Downregulation of major histocompatibility complex class I (MHC-I) molecules on cancer cells represents a major immune evasion tactic of murine and human cancers^31–34^ and, accordingly, enhancement of MHC-I status has been identified as a key target for effective immunotherapy^35–37^. In the present study we have demonstrated that radiation increases MHC-I expression on cancer cell surfaces, enhances susceptibility of normally resistant cancer cells to control by antigen-specific CD8+ T cells and identified a novel mechanism of MHC-I regulation by induction of NLRC5 gene expression.

Differential responses of CD8+ T cells to varying pMHC (peptide-MHC complex) concentration have been observed previously, wherein increased epitope density corresponds with greater responsiveness to IL-2, enhanced proliferation and increased cytotoxic function including cytokine production^38–40^. This phenomenon is better understood in naïve T cells, where high levels of antigen presentation in combination with costimulation and integrin stabilization are required to generate a stable immunological synapse and to cross an activation threshold of T cell receptor (TCR) signaling^40,41^. In activated T cells, pMHC:TCR interactions at the kinapse are much shorter: a single pMHC complex can serially engage with rapidly internalizing TCRs and a CD8+ T cell can exert cytotoxic functions after engaging with as few as 1-3 pMHC complexes per target cell^42,43^. It is clear that higher concentrations of pMHC can engage more TCRs and it has been proposed that serial engagement of the TCR allows increased stability and enhanced signaling within the TCR/pMHC-I/CD8 molecular complex^39^. Functionally, downregulation of MHC-I induced by viral infection can significantly attenuate the ability of CD8+ T cells to kill infected targets^44^. Interestingly, expression of the early activation marker CD69 appears to be independent of epitope density^45^, corresponding with the data presented here demonstrating equivalent expression of Nur77 by naïve T cells cultured with MHC-I^lo^ Panc02SIY100 versus MHC-I^hi^ Panc02SIY100-NLRC5. However, we observed improved IFNγ and TNFα production by activated CD8+ T cells cultured with MHC-I^hi^ Panc02SIY100-NLRC5 cells in line with previous observations that increased epitope density is associated with increased T cell functional activation. Cumulatively, these data suggest that different densities of pMHC can activate different thresholds in T cells for expression of early activation markers, cytolytic degranulation and cytotoxic cytokine release^46^. These experiments support the hypothesis that increased engagement of T cell receptors of effector CD8+ T cells enhances cytotoxic effector functions over those with minimally engaged TCRs. Importantly, in growing tumors the affinity of existing T cells may be sufficient to recognize cancer cells as measured by upregulation of activation markers, but below the critical threshold for effector functions. We interpret these data to suggest that it may be critical for tumors to prevent any significant IFNγ secretion by tumor-specific T cells, since this could result in MHCI upregulation and permit a higher avidity interactions. However, we are studying tumors that successfully grow in immune competent mice, or have become clinically significant in patients, suggesting that some degree of selection has occurred in the cancer cell-host interactions to keep T cell activity below this threshold. This work agrees with findings that tumor downregulation of antigen presentation via MHC-I correlates with checkpoint blockade resistance in human patients^36,37^ and highlights that total loss of MHC-I is not necessary for resistance to T cells.

In this study, we propose a novel mechanism for increasing MHC-I density on cancer cell surfaces induced by radiation. Previous studies of MHC-I regulation by radiation have primarily focused on two distinct mechanisms for induction. One mechanism has highlighted tumor-derived type I interferon (IFN-I) signaling following irradiation as a mechanism of MHC-I induction^22^. IFN-I secretion has been observed following cancer cell irradiation downstream of activation of cGAS/STING signaling^20^, but we found that MHC-I expression on cancer cells following irradiation was not attenuated by knockdown of the IFN-I receptor (IFNAR). Additionally, we observed very little expression of STING in pancreatic cell lines that upregulated MHC-I in response to radiation. Together, these data suggest that in these models MHC-I upregulation is not dependent on IFN-I or STING signaling. Alternatively, another mechanism has proposed that RT-induced protein damage and enhanced mTOR signaling, as a means to facilitate damage repair responses, leads to increased abundance of intracellular peptide pools and enhanced MHC-I loading to the cell surface^47^. We found that genes involved in antigen presentation on MHC-I, including the MHC-I transactivator NLRC5, were upregulated following treatment, but surprisingly that this was not mediated via STING-mediated innate sensing leading to type I IFN signaling (data not shown). To our knowledge this represents a novel mechanism of MHC-I induction on cancer cells by radiation. Together with data showing increased cytokine release by CD8+ T cells cultured with NLRC5-expressing Panc02SIY100, these data support our proposal that induction of NLRC5 by radiation enhances the ability of T cells to functionally respond to cancer cells expressing cognate antigen allowing for tumor regression and clearance.

Our findings here concur with prior observations that tumor control by radiotherapy and checkpoint inhibitors depends on pre-existing immune responses rather than by vaccination by tumor irradiation^1^. In preclinical models, implantation of cancer cells can induce CD8+ T cell priming sufficient to establish T cell memory and can cause spontaneous rejection of immunogenic tumors^1,48–51^; in models where pre-existing anti-tumor immunity is not present, additional vaccination is necessary to permit tumor control by radiation and checkpoint inhibition^52^. While vaccination effects by radiation have been observed here and elsewhere^11^, these data suggest that this response is inadequate to generate sufficiently effective T cells for tumor control. The present study supports an alternate mechanism by which radiation allows CD8+ T cell-mediated control of tumors by genetic enhancement of antigen presentation via MHC-I, thus increasing the “visibility” of cancer cells to pre-existing antigen-matched T cells to enhance cytotoxicity. Importantly, this does not result in tumor cure without the addition of PD-1/PD-L1 blockade. While radiation increases MHCI, it also increases PD-L1 on cancer cells^53^ and other immune cells in the tumor environment^8^, simultaneously reducing the ability of T cells to become activated. PD-1 ligation triggers activation of the phosphatase Shp2 in T cells, which limits costimulatory signaling through CD28^54^. Shp2 dephosphorylation of CD28 therefore increases the overall threshold for T cell activation despite radiation-mediated increases in avidity. Thus, the combination of MHCI increase and PD-1/PD-L1 blockade act together to optimize T cell responses.

NLRC5 was recently described as a master regulator of MHCI expression, with a broad set of target genes including critical elements of the antigen processing and presentation pathway^24^. The effects of NLRC5 on MHCI is broadly comparable to the effects of CIITA as a transactivator of MHCII expression, acting through *cis*-regulatory elements in the proximal promoter^25,55^, and NLRC5 and CIITA represent a distinct subgroup of the NLR family^56^. Notably, while MHCI expression is highly dependent on NLRC5 in many cell types, antigen presenting cells are only slightly affected by loss of NLRC5^57^. Overexpression of NLRC5 in cancer cells dramatically changed their immunogenicity, associated with upregulation of critical antigen processing and presentation components, and improving their recognition by specific T cells^58^. Thus, loss of NLRC5 represents a potential mechanism of immune evasion in cancer cells^27^. It is notable that in our models NLRC5 expression is highly variable between models, but is responsive to treatment with radiation therapy. Since this mechanism is independent of STING and IFNAR1 in the cell, it would appear to not be regulated by classical STAT1-mediated activation of NLRC5 expression. Recently, Burr *et al*. demonstrated that Polycomb Repressive Complex 2 was co-opted to silence MHCI expression in cancer cells in a STAT1-independent manner, and acts in part to repress NLRC5^59^. Thus, it is possible that radiation may affect the epigenetic regulation of NLRC5. However, the precise mechanism of NLRC5 regulation by radiation remains to be determined.

The ability of radiation to modify in-field cancer cells and alleviate αPD-1/PD-L1 blockade resistance highlights the need for comprehensive radiotherapy approaches encompassing the bulk of disease. This perspective supports incorporating combination approaches into earlier stages of disease, either in oligometastatic or locally advanced cancer, where all sites of gross disease are encompassed by the field of radiation.

## Methods

### Animals and cell lines

All experiments were performed in accordance with institutional guidelines and regulations – animal protocols were approved by the Earle A. Chiles Research Institute IACUC (Animal Welfare Assurance No. A3913-01). 5–8 week old C57BL/6 mice (Stock #000664) were purchased from the Jackson Laboratory (Bar Harbor, ME) for use in these experiments. Nur77^GFP^ reporter mice were kindly provided by Dr. Weinberg (Earle A. Chiles Research Institute, Portland, OR)^17^. 2C transgenic mice that express a T cell receptor specific for the the SIYRYYGL peptide presented on H2K_b_ were kindly provided by Dr. Gajewski (University of Chicago, Chicago, IL), bred in-house and crossed with Nur77^GFP^ reporter mice. OT-I transgenic mice that express a T cell receptor specific for the SIINFEKL peptide presented on H2K_b_ were gifted by Dr. Redmond (Earle A. Chiles Research Institute). Pdx-Cre mice (Stock #014647, Jackson Laboratory) were crossed with B6.129S4-Gt(ROSA)26Sor^tm3(CAG-luc)Tyj^/J (Stock #009044, Jackson Laboratory) to generate Pdx-Cre^+^SIY^+^ animals. Leaky expression of luciferase-SIY in these mice results in thymic tolerance to the SIYRYYGL peptide^1^, as has been previously shown with mice with a lung-restricted expression of Cre recombinase^60^. B6.SJL-Ptprc^a^ Pepc^b^/BoyJ mice expressing CD45.1 were obtained from the Jackson Laboratory (Stock#002014) for *in vivo* cytoxicity assays. Survival experiments were performed with 6–8 mice per group. The experiment with SIY tolerant mice was performed with four animals per group.

Cell lines were cultured in RPMI-1640 (HyClone, Fisher Scientific, Hampton, NH) supplemented with 10% heat inactivated fetal bovine serum (Cat#10082147, Thermo Fisher Scientific, Waltham, MA), 2 mM L-glutamine (Cat#SH3003401, HyClone, Fisher), 10 mM HEPES (Cat#HOL06, Caisson Labs, Smithfield, UT), 100 U/mL penicillin-streptomycin (Cat#PSL01, Caisson), 1X non-essential amino acids (Cat#SH3023801, Fisher), 1 mM sodium pyruvate (Cat#PYL01, Caisson). The parental murine pancreatic adenocarcinoma cell line Panc02 was kindly provided by Dr. Woo (Mount Sinai School of Medicine, New York, NY). Panc02 expressing the model antigen SIY was kindly provided by Dr. Weishelbaum (University of Chicago, Chicago, IL), as used previously^1^, and expresses GFP-SIY in approximately 40% of cells. Panc02SIY100 was derived and expanded from a high GFP expressing single clone within Panc02SIY on a BD FACSAria II cell sorter. Panc02SIYneg was similarly derived from a low GFP expressing clone sorted from Panc02SIY on a BD FACSAria II cell sorter (Becton Dickinson, Franklin Lakes, NJ). PK5L1940 was generated from established spontaneous pancreatic tumors in Pdx-Cre^+/−^Kras^(G12D)+/−^Trp53^(R172H)+/−^SIY^+^ as previously described^1^.

### Antibodies and reagents

Viability staining was performed in PBS using Zombie Aqua Fixable Viability Kit (BioLegend, San Diego, CA) for 15 minutes prior to staining with fluorescently-conjugated antibodies for flow cytometry. Monoclonal antibodies were used against: CD3 [17A2], CD4 [RM4-5], CD8α [53-6.7], CD11b [M1/70], CD25 [PC61], CD45.1 [A20], CD45.2 [104], CD69 [H1.2F3], CD90.1 [HIS51], CD274 [MIH5], IFNAR1 [MAR1-5A3], IFNγ [XMG1.2], H-2Kb [AF6-88.5.5.3], TNFα [MP6-XT22], F4/80 (BM8), CD11c (N418), CD90.2 (30-H12), MHC-II (M5/114.14.2), Ly-6C (HK1.4), CD103 (2E9), CD24 (M1/69), CD45 (30-F11). Fluorescently-conjugated MHC-multimer complexes were used as follows: pentamer-SIYRYYGL (pSIY, ProImmune, Sarasota, FL) and tetramer-SIINFEKL (tOva, NIH Tetramer Core, Atlanta, GA).

Immunohistochemistry was performed on Zinc-fixed tumors embedded in paraffin preserved as described previously^61^. Five micron sections were stained with primary αCD3 (SP7, Cat#ab16669, Abcam, Burlingame, CA) diluted in blocking buffer, secondary goat anti-rabbit IgG conjugated to HRP (Cat #AP1879, EMD Millipore, Burlingame, MA), and ImmPACT DAB Peroxidase (HRP) Substrate (Cat#SK-4105, Vector Laboratories, Burlingame, CA). Slides were counterstained with hematoxylin 7211 (Cat#S7439-1, Cardinal Health, Dublin, OH). CD3 infiltration was quantified using Aperio ImageScope (Aperio, Sausalito, CA).

For *in vitro* stimulation of cell lines, recombinant mouse IFN-beta protein (Cat#8234-MB-101/CF, RND Systems, Minneapolis, MN) at a final concentration of 1 × 10^3^ U/mL, recombinant mouse IFN-gamma protein (Cat#14-8311-63, Thermo Fisher) at a final concentration of 20 ng/mL and mammalian 2′3′-cGAMP (Cat#tlrl-nacga23-02, InvivoGen, San Diego, CA) at a final concentration of 25 μg/mL were used. Cancer cells were irradiated before plating by timed exposure to a Cs^137^ source in a Gammacell Elan 3000 (MDS Nordion, Ottawa, ON, CAN).

The *in vivo* cytotoxicity experiment was conducted as described previously^62^. Briefly, wild-type mice were vaccinated with *LmSIY* or *LmOva* seven days prior to injection of congenic splenocytes labelled with CFSE (Cat#C34554, Thermo Fisher) and pulsed with peptide (A&A Labs, San Diego, CA). Six hours later, recipient spleens are harvested and analyzed by flow cytometry.

### Gene editing of cancer cell lines

Panc02SIY100-NLRC5 was derived from Panc02SIY100 cells transfected with a plasmid harboring the sequence for hNLRC5 under the constitutive promoter eukaryotic translation elongation factor 1 alpha (EF-1α) (Cat#pUNO1-hNLRC5, Invivogen, San Diego, CA). Cells with constitutively high expression of MHC-I were isolated by cell sorting using a BD FACSAria II cell sorter.

IFNAR1 knockout Panc02SIY100 and PK5L1940 cell lines were generated using Alt-R S.p. Cas9 Nuclease 3NLS (Cat#192528883, Integrated DNA Technologies, Coralville, IA), Alt-R CRISPR-Cas9 tracrRNA ATTO 550 (Cat#129528884, IDT), Opti-MEM I Reduced Serum Medium (Cat#31985062, Thermo Fisher), Lipofectamine CRISPRMAX Cas9 Transfection Reagent (Cat#CMAX00015, Thermo Fisher Scientific, Waltham, MA) and predesigned Alt-R CRISPR-Cas9 crRNA guide RNAs (IFNAR1 sequence: 5′-TCAGTTACACCATACGAATC-3′). Three gRNAs were tested for each target and pure populations were isolated from single cells (Panc02SIY100) or five cells (PK5L1940) based on ability to upregulate MHC-I in response to cytokine stimulation using a BD FACSAria II cell sorter.

### Listeria monocytogenes vaccination

*ActA* deleted (*ΔactA*) *Listeria monocytogenes* (*Lm*) strains used for vaccination were engineered to express SIYRYYGL peptide (*LmSIY*) or SIINFEKL peptide of ovalbumin (*LmOva*) cloned in frame with the ActA N-terminal fragment. Bacteria were grown in brain-heart infusion broth, washed twice in PBS and administered by retro-orbital injection at a dose of 1 × 10^7^ CFU in 100 μL total volume. Effective vaccination was confirmed seven days later by MHC-multimer binding of peripheral blood and as previously described^63^.

### Immunotherapy and radiation therapy of tumors

Tumors were inoculated at a dose of 5 × 10^6^ for Panc02SIY tumors, 10 × 10^6^ for Panc02SIY100 and 10 × 10^6^ for Panc02SIY100-IFNAR1^ko^ tumors. Tumor size was determined via caliper measurements of the longest length x the longest perpendicular width. Survival endpoint was defined as tumor size greater than or equal to 150 mm^2^ or when the mouse appeared moribund.

For *in vivo* experiments, 12 Gy of CT-guided radiation was administered to tumor isocenters using a Small Animal Research Radiation Platform (SARRP) (Xstrahl, Suwanee, GA) and Murislice software (Xstrahl), 14 days after tumor implantation. 250 μg per dose αPD-L1 checkpoint blockade (Cat#BE101, BioXCell, West Lebanon, NH) was administered intraperitoneally at day 7, 14 and 21 post tumor implantation. Therapeutic *LmSIY* was administered at day 14 following tumor challenge to coincide with radiation controls.

### Tumor analysis

For flow cytometric analysis of tumor-infiltrating cells, tumors harvested seven days after treatment were chopped into small fragments and dissociated in a solution of 250 U/mL collagenase IV (Worthington Biochemical Corporation, Lakewood, NJ) and 30 U/mL DNase (Millipore Sigma, St Louis, MO) using a GentleMACS tissue dissociator (Miltenyi Biotec, Auburn, CA). After 30 minutes incubation at 37 °C, the digest was quenched in RPMI-1640 (Cat#SH30027LS, Fisher) supplemented with 10% FBS (Cat#16000069, Thermo Fisher) and 2 mM EDTA (Cat#324504, Millipore Sigma) and strained through 100 μM and 40 μM cell strainers. Filtered cells were rinsed in cold PBS twice prior to counting and staining for analysis on a BD LSR II flow cytometer (Becton Dickinson). For analysis of infiltrates of wild-type versus IFNAR1^ko^ tumors, tumors were weighed and minced into small fragments, then transferred into C tubes from Miltenyi Biotec containing enzyme digest mix and the tissue was dissociated using a GentleMACS (Miltenyi Biotech). This was followed by incubation at 37 °C for 30 min with agitation and enzymatic reactions were quenched using ice cold RPMI containing 10% FBS and 2 mM EDTA.

For staining, 2 × 10^6^ cells were stained with Zombie Aqua Viability Dye from BioLegend (#423102) in PBS for 10 min on ice, then Fc receptors were blocked with a-CD16/CD32 antibodies from BD Biosciences (2.4G2) for an additional 10 min. After centrifugation, the supernatant was removed and cell were stained with a surface antibody cocktail containing in FACS buffer (PBS, 2 mM EDTA, 2% FBS) and Brilliant Stain Buffer Plus from BD Biosciences (#566385) for 20 min on ice. After surface staining, cells were washed in FACS buffer and fixed for 20 min on ice with Fixation/Permeabilization Buffer from BD Biosciences (#554722). All samples were resuspended in FACS buffer and acquired on a BD Fortessa flow cytometer. Data were analyzed using FlowJo software from Tree Star, v10.5.

### *In vitro* T cell coculture assays

Prior to coculture with cancer cells, CD8+ T cells were activated *in vivo* with *Listeria* vaccines or harvested as splenocytes from naïve animals and activated *in vitro* with αCD3ε (Cat#BE0001-1, BioXCell) and αCD28 (Cat#BE0015-1) at a final concentration of 10 μg/mL each. For *in vitro* activation, after 48 hours, cells were rinsed with 10% complete RPMI supplemented with β-mercaptoethanol (βME) (Cat#21985023, Gibco, Thermo Fisher) and replated with 60 IU/mL human recombinant IL-2 (Chiron) for three days. Regardless of route of activation, CD8+ T cells were purified from splenocytes using a CD8α+ T cell negative isolation kit (Cat#130-104-075, Miltenyi) and counted prior to coculture.

Cancer cell growth was monitored using an Incucyte (Sartorius, Goettingen, Germany) and Zoom software (Incucyte, Sartorius). Briefly, cancer cells were plated in the presence of cytokines or following *in vitro* irradiation. After 24 hours, adherent cells were rinsed twice in 10% complete RPMI supplemented with βME. Purified CD8+ T cells from *Lm*-vaccinated animals were added to cancer cell cultures at a 100:1 effector:target ratio; T cells from transgenic 2C or OT-I animals were plated at 5:1. Non-adherent cells were allowed to settle for 15 minutes at room temperature prior to hourly tracking in the Incucyte, housed at 36.5 °C/5%CO_2_, until untreated cells reached confluence. In order to isolate cancer cells from T cells for analysis, endogenous green fluorescence was used as a proxy for cell confluence.

Cytokine production by T cells during coculture was determined after four-hour incubation with brefeldin A (Cat#B7450, Thermo Fisher). Non-adherent cells were harvested, surface stained and permeablized using BD cytofix/cytoperm fixation and permeabilization solution (Cat#554655, Becton Dickinson), then frozen at −80 °C overnight. The next day, cells were thawed and rinsed twice with perm/wash buffer (Cat#554723, Becton Dickinson) and stained for intracellular cytokines.

### Immunoblotting

Briefly, cells from pancreatic cell lines were washed twice with PBS and lysed in Pierce RIPA Buffer (Cat#8990, Thermo) supplemented with Halt Protease and Phosphatase Inhibitor Cocktail 100×(Cat#78440, Thermo). Protein concentrations were quantified using Pierce BCA Protein Assay Kit (Cat#23225, Thermo). Samples were denatured at 95 °C in XT Sample Buffer 4×(Cat#1610791, Bio-Rad, Hercules, CA) and loaded onto 4–12% Criterion XT Bis-Tris Protein Gels (Cat#345-0124, Bio-Rad). Proteins were transferred onto PVDF Transfer Membrane (Cat#88518, Thermo) and probed for STING (Cat#13647S, Cell Signaling Technology, Danvers, MA), IRF3 (Cat#4302S, Cell Signaling), STAT1 (Cat#9172S, Cell Signaling), p-STAT1 (Y701) (Cat#7649S, Cell Signaling) and GAPDH (Cat#2118S, Cell Signaling). HRP-conjugated goat anti-rabbit IgG (Cat#31460, Invitrogen, Carlsbad, CA) was used as a secondary antibody. Proteins were visualized using SuperSignal West Pico PLUS Chemiluminescent Substrate (Cat#34580, Thermo).

### RNAseq of human pancreatic tumors

Analysis of patient pancreatic tumors were approved under Providence IRB 10-037. We restricted our analysis to adult patients (18–100 years of age) who underwent up-front resection for pancreatic ductal adenocarcinoma^64^. We excluded patients who received neoadjuvant therapy or had other cancer histologies. Demographics are outlined in Table 1. RNASeq was performed on archived paraffin-embedded tissue specimens from 35 patients. The FFPE tissue sections were deparaffinized using Envirene (Hardy Diagnostics) followed by RNA extraction and purification using the Qiagen AllPrep DNA/RNA FFPE kit. 85 ng of input RNA was used to prepare sequencing libraries using the Illumina TruSeq RNA Exome kit. Sequencing of the RNA Exome libraries was performed on the Illumina HiSeq. 4000 instrument at 2 × 75 read paired end configuration. Transcripts were quantified using salmon-v.0.11.2^65^.

Gene expression analyses were performed using BRB-ArrayTools developed by Dr. Richard Simon and the BRB-ArrayTools Development Team.

### Statistics

Data was analyzed and graphed using FlowJo (Tree Star, Ashland, OR) and Prism (GraphPad Software, La Jolla, CA). Individual data sets were compared using Welch’s T tests. Growth curves and analysis across multiple groups were analyzed using ANOVA (two-way and one-way, respectively). Kaplan Meier survival curves were compared using log-rank tests.

## Supplementary information

Supplementary information.
