# Supplementary material for: Transcriptional Upregulation of NLRC5 by Radiation Drives STING- and Interferon-Independent MHC-I Expression on Cancer Cells and T Cell Cytotoxicity
Source: Sci Rep. 2020 Apr 30;10:7376. doi: 10.1038/s41598-020-64408-3 (PMC7193601; doi:10.1038/s41598-020-64408-3)
Supplement: Supplementary file 1 — Supplementary information. [file 41598_2020_64408_MOESM1_ESM.pdf]

Supplementary data.

Supplementary Figure 1

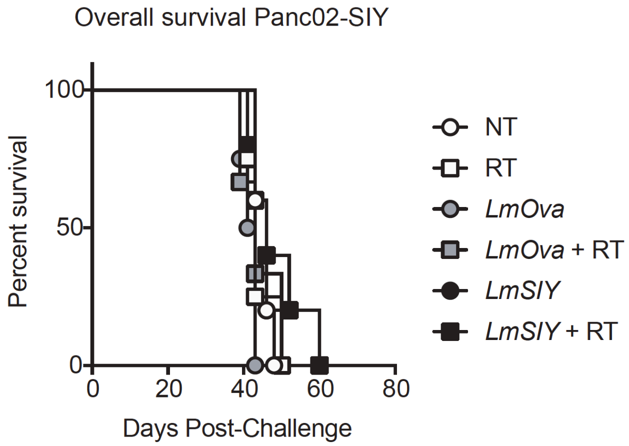

C57BL/6 mice bearing Panc02-SIY were vaccinated with LmOva or LmSIY on day 17 following tumor challenge and irradiated on d20. Mice were followed for overall survival.

Supplementary Figure 2

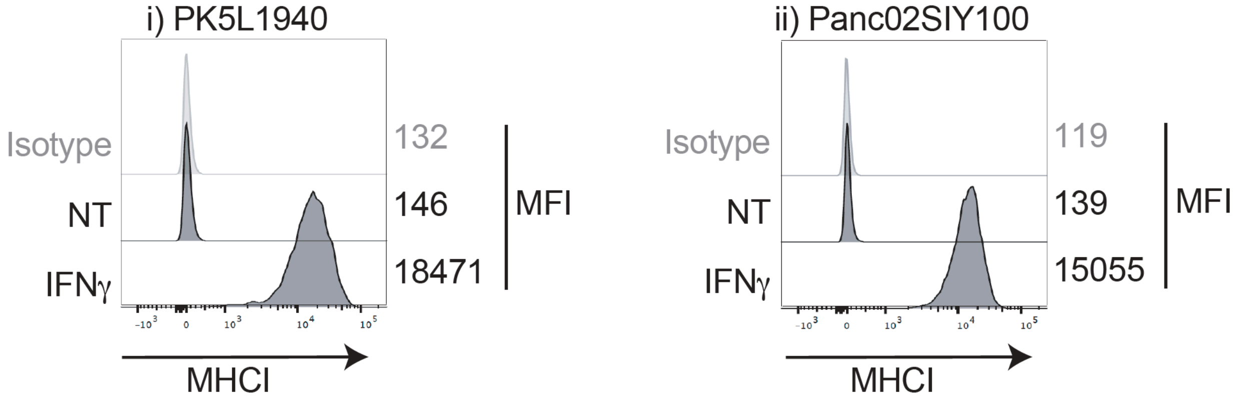

Flow cytometry of untreated or IFNg-treated i) PK5L1940 or ii) Panc02SIY100 with isotype control antibodies or antibodies to MHCI. Numbers indicate MFI of stained cells.

Supplementary Figure 3

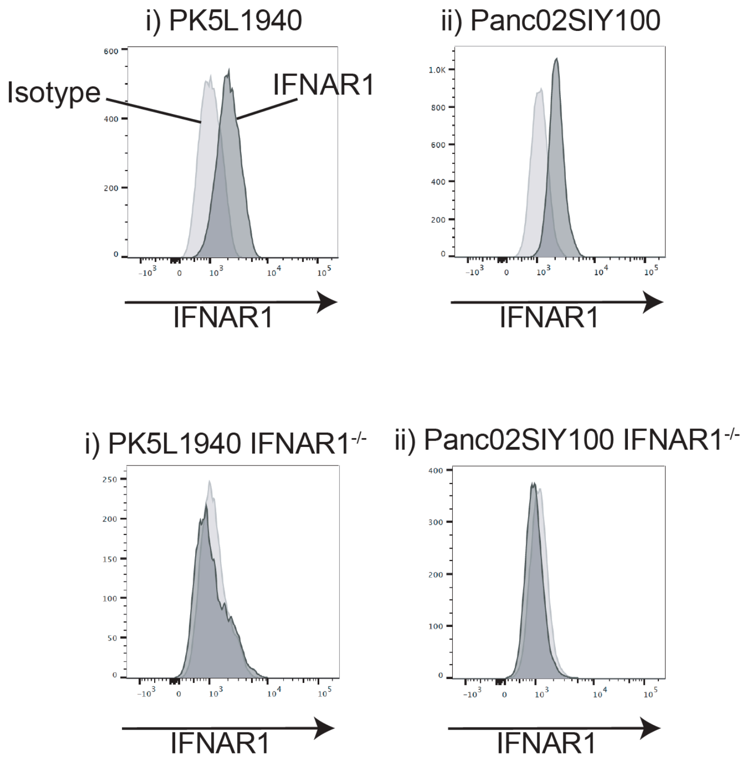

Flow cytometry of i) PK5L1940 or ii) Panc02SIY100 with isotype control antibodies or antibodies to IFNAR1. Top panels show wild-type cells, bottom panels show IFNAR1^{ko} cells.

Supplementary Figure 4

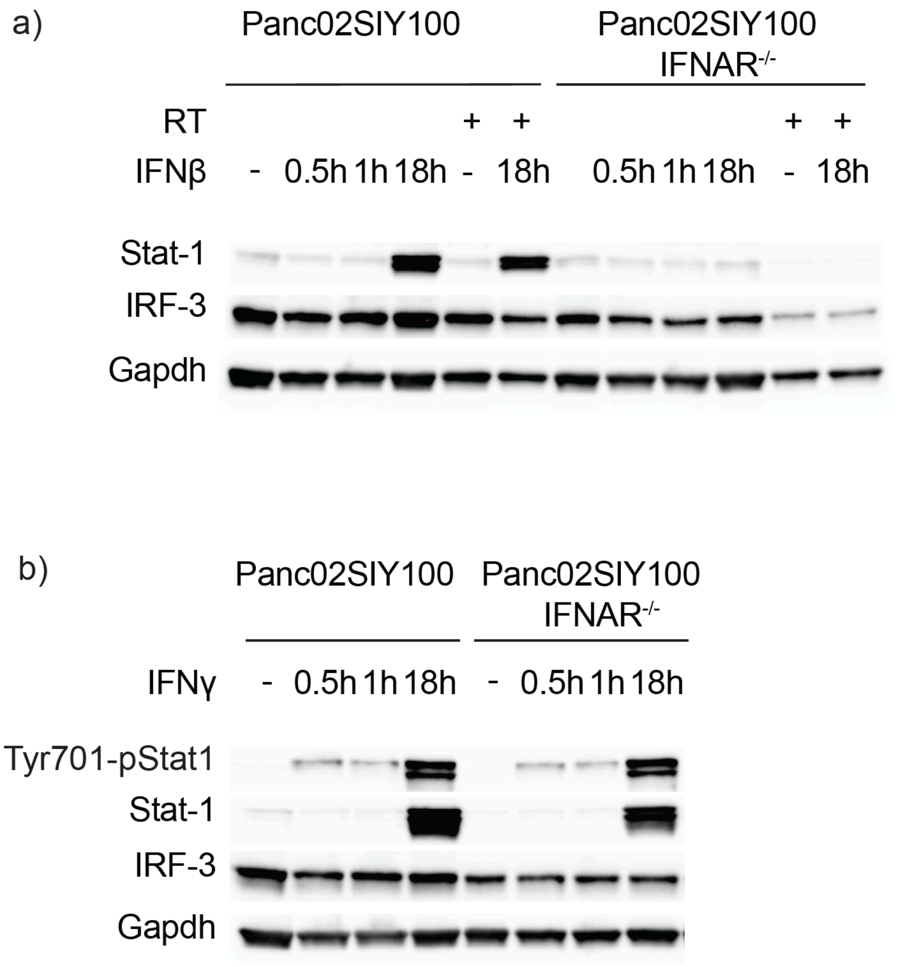

Western blot of Panc02SIY100 and Panc02SIY100IFNAR1^{ko} following treatment with a) type I IFN and/or RT or b) IFNg over a time course.

Supplementary Figure 5

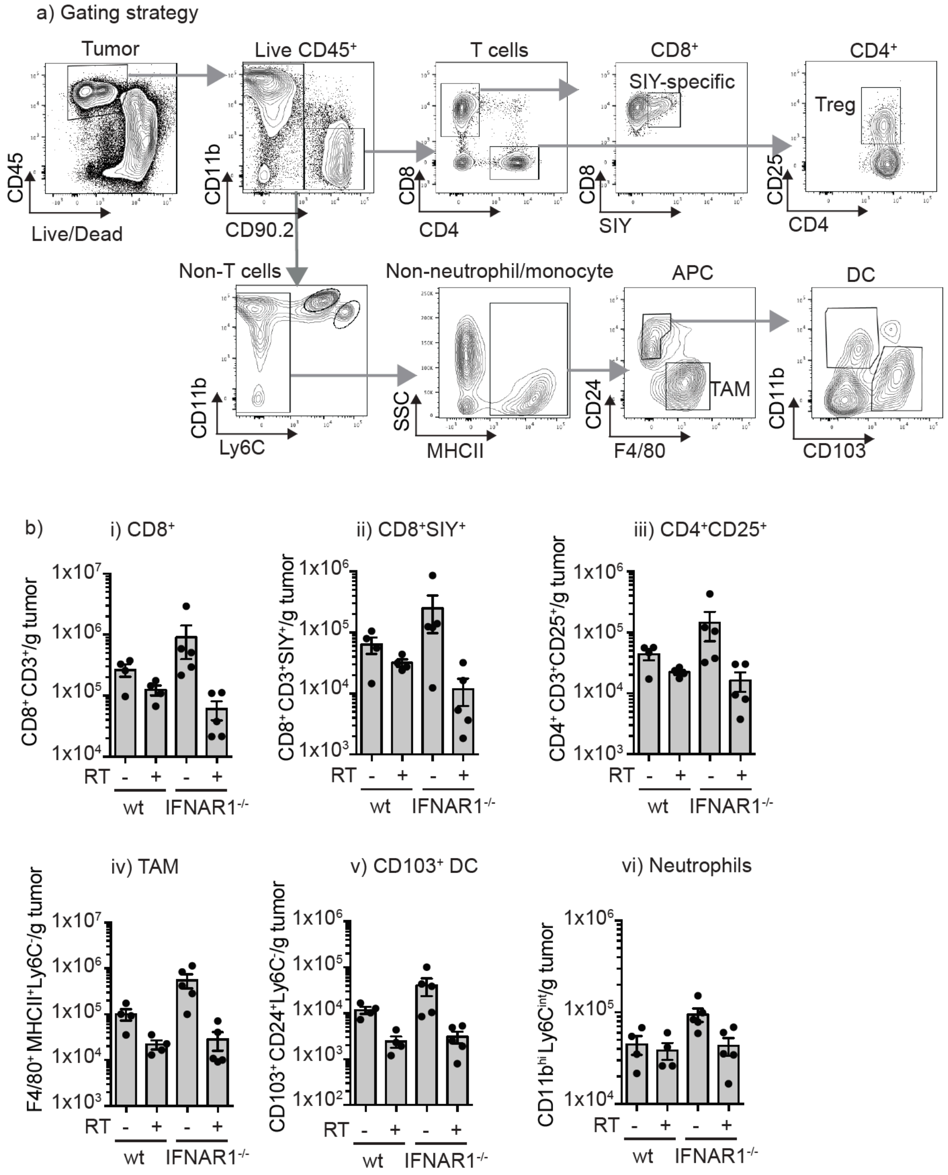

Flow cytometry of Panc02SIY100 and Panc02SIY100IFNAR1^{ko} tumors left untreated or treated with 12Gy RT. a) gating strategy b) Major populations of infiltrating cells represented as cells/mg tumor.

Supplementary Figure 6

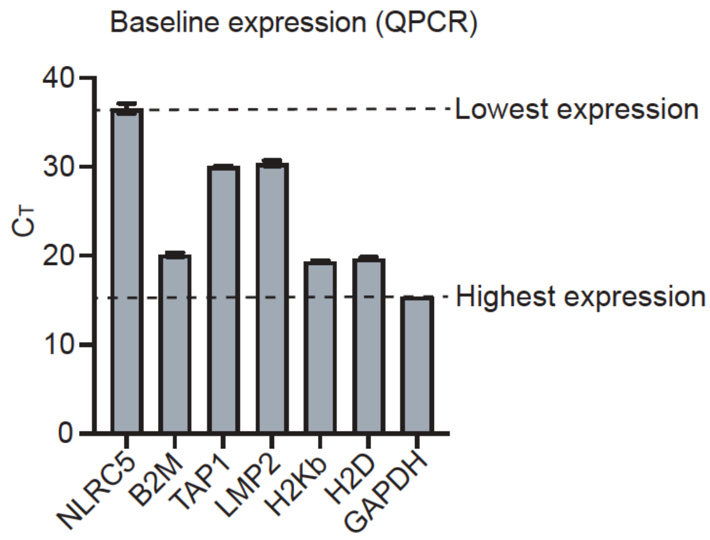

qPCR analysis of antigen processing and presentation components in Panc02SIY100 cancer cells at baseline. Data is represented where fewer cycles indicates a higher abundance of transcript.

Supplementary Figure 7

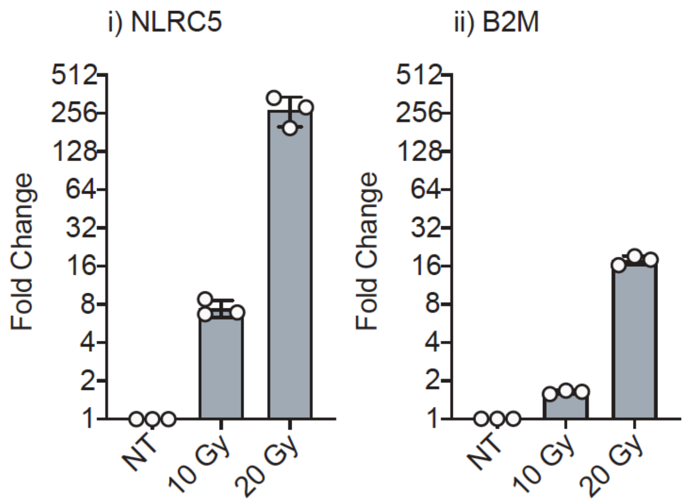

qPCR analysis of NLRC5 and B2M in Panc02SIY100IFNAR1^{ko} cancer following treatment with 10 or 20Gy RT. Data is represented as fold change versus untreated cells.

Supplemental Table 1.

Correlated Gene	Cytoband	Spearman's Correlation	p-Value	q-Value
**HLA-F**	**6p22.1**	**0.8571073**	**2.71E-52**	**5.41E-48**
**TAP1**	**6p21.32**	**0.82512422**	**2.91E-45**	**2.91E-41**
**HLA-C**	**6p21.33**	**0.81705866**	**1.03E-43**	**6.87E-40**
**HLA-A**	**6p22.1**	**0.79311733**	**1.57E-39**	**7.84E-36**
HCP5	6p21.33	0.75951334	1.64E-34	6.55E-31
PSMB9	6p21.32	0.75241945	1.48E-33	4.93E-30
PSMB8	6p21.32	0.74837477	5.02E-33	1.43E-29
**HLA-E**	**6p22.1**	**0.7292572**	**1.19E-30**	**2.98E-27**
**HLA-H**	**6p22.1**	**0.68336767**	**1.08E-25**	**2.39E-22**
**B2M**	**15q21.1**	**0.67768044**	**3.83E-25**	**7.65E-22**
HCG26	6p21.33	0.65615207	3.66E-23	6.64E-20
CD74	5q33.1	0.62363446	1.84E-20	3.06E-17
IFI35	17q21.31	0.60507739	4.67E-19	7.18E-16
NFKBIE	6p21.1	0.60012379	1.07E-18	1.53E-15
**IRF1**	**5q31.1**	**0.59426776**	**2.80E-18**	**3.73E-15**
**NLRC5**	**16q13**	**0.59135489**	**4.48E-18**	**5.59E-15**
**TAP2**	**6p21.32**	**0.58598795**	**1.05E-17**	**1.24E-14**
TAPBP	6p21.32	0.58503142	1.22E-17	1.36E-14
UBE2L6	11q12.1	0.58457696	1.31E-17	1.38E-14
TYMP	22q13.33	0.58276346	1.75E-17	1.74E-14
ZBP1	20q13.31	0.57975754	2.78E-17	2.65E-14
IL4I1	19q13.33	0.57133057	1.00E-16	8.98E-14
SLC15A3	11q12.2	0.57094103	1.07E-16	8.98E-14
LGALS9	17q11.2	0.57085663	1.08E-16	8.98E-14

Top 25 genes with RNA expression most correlated with HLA-B mRNA expression according to cBioportal analysis of TCGA Pancreatic Adenocarcinoma PancCancer Atlas.
